# Impact of Mild Traumatic Brain Injury (mTBI) on CYP2D6 Activity and the Restorative Effects of Melatonin and Vitamin C Supplementation

**DOI:** 10.5812/ijpr-164903

**Published:** 2026-02-08

**Authors:** Kaveh Berenjian, Ziba Gholipour, Mohammadreza Khoshayand, Mohammad Sharifzadeh, Mohammadreza Rouini, Yalda Hosseinzadeh Ardakani

**Affiliations:** 1 Biopharmaceutics and Pharmacokinetic Division, Department of Pharmaceutics, Faculty of Pharmacy, Tehran University of Medical Sciences, Tehran, Iran; 2 Department of Drug and Food Control, Faculty of Pharmacy, Tehran University of Medical Sciences, Tehran, Iran; 3 Department of Toxicology and Pharmacology, Faculty of Pharmacy, Tehran University of Medical Sciences, Tehran, Iran

**Keywords:** Mild Traumatic Brain Injury, Cytochrome P-450 2D6, Metabolic Ratio, Melatonin, Vitamin C, Pharmacokinetics

## Abstract

**Background:**

Mild traumatic brain injury (mTBI) may disrupt hepatic cytochrome P-450 enzyme activity, potentially altering drug pharmacokinetics.

**Objectives:**

This study investigated the impact of mTBI on CYP2D6 activity and the restorative effects of melatonin and vitamin C supplementation.

**Methods:**

Mild traumatic brain injury was induced in rats using a modified weight-drop model, which were then assigned to treatment or control groups. CYP2D6 activity was assessed at multiple time points post-injury using metabolic ratios of tramadol and mirtazapine through isolated liver perfusion. All experimental procedures were conducted with the investigator blinded to group assignments.

**Results:**

Mild traumatic brain injury significantly suppressed CYP2D6 activity on day 3, with partial recovery by day 7. While tramadol metabolism normalized by day 28, mirtazapine metabolism remained impaired in non-supplemented groups. Supplementation accelerated CYP2D6 recovery, showing statistically significant change by day 7. By day 28, supplemented groups exhibited metabolic ratios surpassing baseline levels.

**Conclusions:**

Mild traumatic brain injury induces transient suppression of CYP2D6 activity, which was attenuated in antioxidant-treated groups in this preclinical model. Further studies are needed to explore the clinical relevance of these findings.

## 1. Background

Mild traumatic brain injury (mTBI) is a prevalent neurological condition that can lead to a range of cognitive, emotional, and physiological disturbances (1, 2). While often considered less severe than moderate or severe traumatic brain injury, mTBI can still induce significant alterations in brain function and systemic physiology, including the regulation of hepatic drug-metabolizing enzymes (3, 4).

Cytochrome P450 (CYP) enzymes, particularly CYP2D6, play a critical role in the metabolism of many clinically relevant drugs. Evidence suggests that inflammatory responses triggered by brain injury can transiently suppress CYP2D6 activity, potentially altering drug pharmacokinetics (5-7). Understanding the dynamics of CYP2D6 modulation following mTBI is therefore crucial for predicting drug metabolism in affected individuals.

Antioxidant supplementation has been proposed as a potential strategy to mitigate oxidative stress and support recovery after neurological injury (8-10). In particular, melatonin and vitamin C are known for their neuroprotective and antioxidative properties. However, their effects on hepatic CYP2D6 activity following mTBI remain unclear (10-12).

In this study, we hypothesized that supplementation with melatonin and vitamin C may attenuate the suppressive effects of mTBI on CYP2D6 activity. By investigating these preclinical mechanisms, we aim to provide foundational insights that could inform future therapeutic strategies while emphasizing the exploratory nature of this research.

## 2. Objectives

This study aimed to investigate the effect of mTBI on hepatic CYP2D6 enzyme activity and subsequent drug metabolism in an experimental animal model. Furthermore, it sought to evaluate whether melatonin and vitamin C supplementation could mitigate the mTBI-induced alterations in CYP2D6 activity. We hypothesized that antioxidant supplementation would promote faster enzymatic recovery following mTBI.

## 3. Methods

### 3.1. Chemicals

The study utilized chemicals and reagents from various suppliers. Racemic tramadol and its metabolite M1 were provided by Grunenthal (Germany), while injectable vitamin C was obtained from Caspian Tamin Pharmaceutical Co. (Iran). Mirtazapine (MRZ), its 8-OH metabolite, and NDES metabolite were supplied by Mario Georgi (Italy). HPLC-grade solvents, analytical-grade salts, and melatonin were sourced from Merck (Germany) and Sigma-Aldrich (USA). Purified water was obtained from a Millipore system. The Krebs-Henseleit buffer was freshly prepared and equilibrated with an oxygen-carbon dioxide mixture. All other chemicals were of analytical or HPLC grade.

### 3.2. Experimental Protocol

Healthy male Wistar rats (180 - 200 g) were housed under controlled conditions (12-hour light/dark cycle, 25 ± 2° C, 55% relative humidity) with unrestricted access to food and water. Environmental enrichment was provided in accordance with ARRIVE guidelines, including nesting material, cardboard tunnels, and social housing (3 rats per cage) to promote natural exploratory behavior and reduce stress.

All procedures conformed to the National Institutes of Health (NIH) Guide for the Care and Use of Laboratory Animals and were approved by the Ethics Committee of Tehran University of Medical Sciences (ethical code: IR.TUMS.TIPS.REC.1400.002). Animals were randomly assigned to groups using a computer-generated randomization sequence to minimize bias.

#### 3.2.1. Randomisation and Blinding

Animals were allocated to experimental groups using a computer-generated random sequence. The allocation sequence was generated by an independent researcher who was not involved in animal handling, sample collection, or outcome assessment. Group assignments were encoded (numeric ID labels) and provided to the animal facility staff for cage assignment; investigators responsible for surgical procedures and post-operative care were aware only of cage IDs, not group identity.

For sample processing and biochemical assays, tissue and plasma samples were relabelled with anonymised codes by a technician not involved in downstream analyses to ensure that laboratory personnel remained blinded to group assignment. Activity assays and biomarker measurements were performed according to pre-specified protocols without access to the allocation key. Data files were compiled with coded identifiers and provided to the statistician who performed the primary analyses while blinded to group identities; the allocation key was only revealed after completion of the primary statistical analysis.

Any deviations from the randomisation or blinding procedures (e.g., unexpected exclusions for humane endpoints) were documented contemporaneously and are reported in the Supplementary File.

#### 3.2.2. Experimental Design for Induction of Mild Traumatic Brain Injury

An mTBI model was established using a modified weight-drop method optimized via a central composite design (CCD) approach. The rationale for using CCD was to systematically identify and balance the critical parameters influencing the severity and reproducibility of injury (e.g., drop height, impact force, and protective conditions) while minimizing the number of animals required. This statistical optimization allowed refinement of the mTBI protocol to achieve consistent, mild injuries in line with prior literature (5).

On postnatal day 45 (P45), rats were anesthetized with isoflurane and positioned on an aluminum foil stage above a sponge pad. A weight was dropped through a PVC guide tube to induce mTBI, ensuring a single impact per animal. Following injury, lidocaine was applied locally, and rats were kept on a heating pad until recovery before being returned to their enriched cages.

Animal body weights were monitored daily post-injury, and no significant weight loss or signs of distress were observed, indicating adherence to humane endpoints and animal welfare standards.

#### 3.2.3. Behavioral Testing

Behavioral tests were conducted to evaluate neurological and functional outcomes following mTBI. Three standard assays — the neurological severity score (NSS), beam walk test, and open field test — were used to confirm successful mTBI induction. These tests collectively assess motor coordination, anxiety, and general neurological function (13).

##### 3.2.3.1. Neurological Severity Score

The ten-point NSS was used to quantify post-injury neurological impairment. The score consists of ten tasks assessing reflexes, motor skills, and alertness; one point is assigned for each failed task. Scores were recorded at 1 and 18 hours after injury by two blinded investigators, with higher scores indicating greater neurological deficit (14).

##### 3.2.3.2. Beam Walk Test

Twenty-four hours post-injury, rats performed the beam walk test on a 165-cm tapered beam. The number of hindlimb slips and the time to traverse the beam were measured to evaluate balance and coordination. Trials were video recorded and analyzed by blinded observers.

##### 3.2.3.3. Open Field Test

Forty-eight hours after injury, locomotor and exploratory behaviors were assessed in a 60 × 60 × 40 cm open field for 300 seconds using an automated tracking system (EthoVision XT^®^, Noldus, Netherlands). Total distance, velocity, and rearing frequency were analyzed as indicators of locomotion and anxiety.

#### 3.2.4. Liver Perfusion Method

Rats were anesthetized with ketamine (75 mg/kg) and xylazine (15 mg/kg). The portal vein and inferior vena cava were cannulated, while the bile duct and superior vena cava were ligated. The liver was perfused with freshly prepared Krebs-Henseleit buffer containing tramadol and/or mirtazapine at controlled flow (10 mL/min) and temperature (25° C). Samples were collected at specified intervals, centrifuged, and stored at -70° C. Liver viability was verified by monitoring AST and ALT levels spectrophotometrically, and inflammation was assessed via serum interleukin-6 (IL-6) using a rat-specific ELISA kit. All biochemical analyses were performed under blinded conditions.

#### 3.2.5. Supplement Doses and Duration

The selected doses of melatonin (10 mg/kg) and vitamin C (100 mg/kg) and the 28-day administration period were based on prior preclinical studies demonstrating antioxidative and neuroprotective efficacy in traumatic brain injury (TBI) and hepatic injury models (14). The 28-day treatment window captured both acute/subacute dysfunction and recovery phases of CYP2D6 activity (15, 16).

#### 3.2.6. Chromatographic Conditions

Quantification of tramadol, mirtazapine, and their metabolites was performed by HPLC with fluorescence detection, following our previous validated protocols (17, 18). Briefly, chromatographic separation used Chromolith™ RP-18e or RP-8e columns, appropriate methanol-water or phosphate buffer–acetonitrile mobile phases, and detection wavelengths optimized for each compound. Data were analyzed using ChromGate software.

#### 3.2.7. CYP2D6 Activity Assessment

CYP2D6 activity was estimated by calculating the metabolic ratio (metabolite/parent drug) for tramadol and mirtazapine. The average of the final three steady-state sampling points during liver perfusion was used as the representative metabolic ratio for statistical comparisons.

#### 3.2.8. Statistical Analysis

Sample size was determined a priori using G*Power (effect size = 1.5, α = 0.05, power = 0.8), requiring a minimum of 3 - 5 animals per group. Data are expressed as mean ± SD. Two-way ANOVA followed by Tukey’s post-hoc test was used for group and time comparisons.

Unpaired *t*-tests were applied only for pre-specified, independent pairwise comparisons (e.g., day-specific control vs treatment), where data distributions were confirmed normal and groups were independent. This approach aligns with prior analytical frameworks for similar preclinical designs and was limited to avoid inflation of Type I error. All analyses were conducted using GraphPad Prism 9.0, with significance set at P < 0.05.

Given the small sample size for certain measurements (n = 3), all primary outcomes are reported with 95% confidence intervals (CI) to indicate the precision of estimated means. Post-hoc power calculations were conducted to assess the likelihood of detecting statistically significant differences given the sample size; these results are interpreted with caution in the discussion.

## 4. Results

### 4.1. Initial Index

Due to the requirement for the use of a protective cap to prevent skull fractures and severe cranial injuries, groups lacking such protective equipment were excluded from the study. In the next phase, the target animal population consisted of juveniles. Therefore, based on epidemiological studies, juvenile rats weighing between 180 and 210 grams — representing the predominant weight range — were selected.

To optimize the selection, the NSS test was conducted on the remaining five groups. The aim was to identify groups with mild to moderate injury severity, defined by a score range of 3 to 5, which serves as the threshold between mild to moderate injury levels.

Ultimately, block 1 was selected as the preferred group for continuation of the study. Additionally, a rapid complementary test assessing time to right was utilized to validate the data.

### 4.2. Behavioral Testing

#### 4.2.1. Neurological Severity Score Test

In this study, the NSS was evaluated at 1 hour and 18 hours following mTBI induction. The results showed a significant increase in NSS at 1 hour post-injury (4.07 ± 0.593) compared to 18 hours post-injury (3.67 ± 0.488), as determined by two-way ANOVA (factor A: Injury, factor B: Time) followed by Tukey's post-hoc test [F(1, 20) = 5.432, P < 0.05 for time], with both time points demonstrating significant differences from the control group (1.33 ± 0.488, P < 0.05). Supplement therapy significantly reduced NSS at both time points compared to non-supplemented groups (3.20 ± 0.414 vs. 3.67 ± 0.488 at 1 hour, and 3.40 ± 0.507 vs. 4.07 ± 0.594 at 18 hours, P < 0.05), as revealed by the significant interaction effect [F(1, 20) = 8.765, P < 0.01] and post-hoc analysis. This interaction corresponded to a large effect (partial η² = 0.44). Supplement-related reductions on days 3 and 7 showed medium effect sizes (Cohen’s d = 0.62 - 0.78).

**Figure 1.**
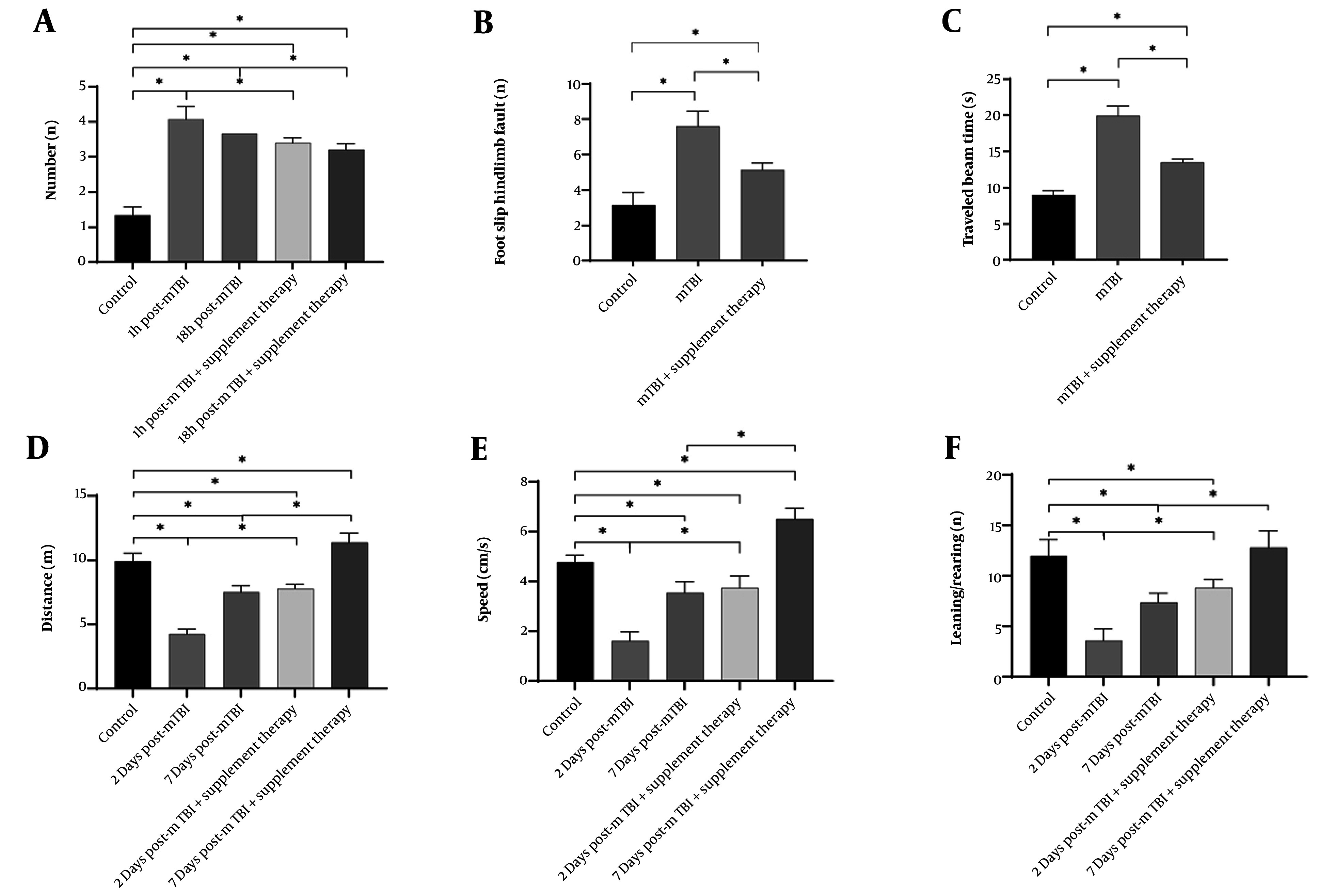
Graphical representation of different behavioral test results by sham and mild traumatic brain injury (mTBI) animals (n = 5, mean ± SD): A, the average number of neurological severity score (NSS); B, the average number of hind-leg foot-slips; C, the average traveled beam time; D, the average distance traveled; E, the average travel speed; F, the number of learning/rearing (* P < 0.05 when compared to the control group).

These findings confirm the successful induction of mTBI and suggest that supplement therapy may reduce neurological deficits following mTBI (Figure 1A). 

#### 4.2.2. Beam Walk Test: Assessment of Motor Function Deficits After Mild Traumatic Brain Injury

To evaluate motor coordination, balance, and fine motor control following mTBI induction, the beam walk test was conducted. Analysis of hind limb foot slips and beam traversal time, performed 24 hours after mTBI, revealed significant motor deficits in the mTBI group compared to the control group. The average number of hind limb foot slips was 7.60 ± 1.24 in the mTBI group, significantly higher than the control group’s 3.13 ± 0.92 (P < 0.001, unpaired *t*-test, t(14) = 9.123). Similarly, the mean beam traversal time was significantly longer in the mTBI group (19.9 ± 2.04 s) compared to the control group (9.00 ± 0.92 s, P < 0.001, unpaired *t*-test, t(14) = 12.456). These results, indicating significant motor coordination deficits and balance impairments in the mTBI group compared to the control group, further confirm the successful induction of mTBI (Figure 1B and C).

#### 4.2.3. Open Field Test: Assessment of Locomotor Activity

The open field test, conducted two days after mTBI induction, revealed significant reductions in locomotor activity. The mTBI group traveled an average distance of 4.21 ± 0.41 m, compared to 9.93 ± 0.62 m for the control group (P < 0.001, unpaired *t*-test, t(14) = 15.789). Similarly, travel speed was significantly lower in the mTBI group, averaging 1.62 ± 0.36 cm/s, in contrast to 4.80 ± 0.28 cm/s in the control group (P < 0.001, unpaired *t*-test, t(14) = 18.234). Rearing behavior also declined significantly, with the mTBI group recording values of 3.6 ± 1.0 on day 2 and 7.4 ± 0.8 on day 7, compared to the control group’s mean of 12.0 ± 1.4 (P < 0.001 for both days, two-way ANOVA, main effect of injury F(1, 40) = 125.678, P < 0.0001). Notably, daily supplementation with vitamin C and melatonin for two days significantly improved locomotor outcomes in the mTBI group. The supplemented group traveled an average distance of 7.76 ± 0.33 m, compared to 4.21 ± 0.41 m in the unsupplemented mTBI group (P < 0.01, unpaired *t*-test, t(10) = 7.891). Travel speed also increased in the supplemented group to 3.74 ± 0.49 cm/s, compared to 1.62 ± 0.36 cm/s in the unsupplemented group (P < 0.01, unpaired *t*-test, t(10) = 8.543). Additionally, rearing behavior improved significantly in the supplemented group, reaching 8.8 ± 0.8 compared to 3.6 ± 1.0 in the unsupplemented group (P < 0.01, unpaired *t*-test, t(10) = 6.789). These findings confirm the successful induction of mTBI in the rats and highlight locomotor activity as one of several indicators of neurological impairment. Moreover, the results demonstrate that supplementation with vitamin C and melatonin significantly mitigates mTBI-related neurological damage, underscoring their potential therapeutic value in post-TBI recovery (Figure 1D, E and F).

### 4.3. Interleukin-6 Post-Traumatic Changes in Interleukin-6 Levels and the Impact of Supplementation

Serum IL-6 levels were analyzed using two-way ANOVA (factor A: Treatment, factor B: Time), which revealed a significant interaction [F(1, 20) = 15.432, P < 0.001], indicating that the effect of supplementation depended on the time point.

**Figure 2.**
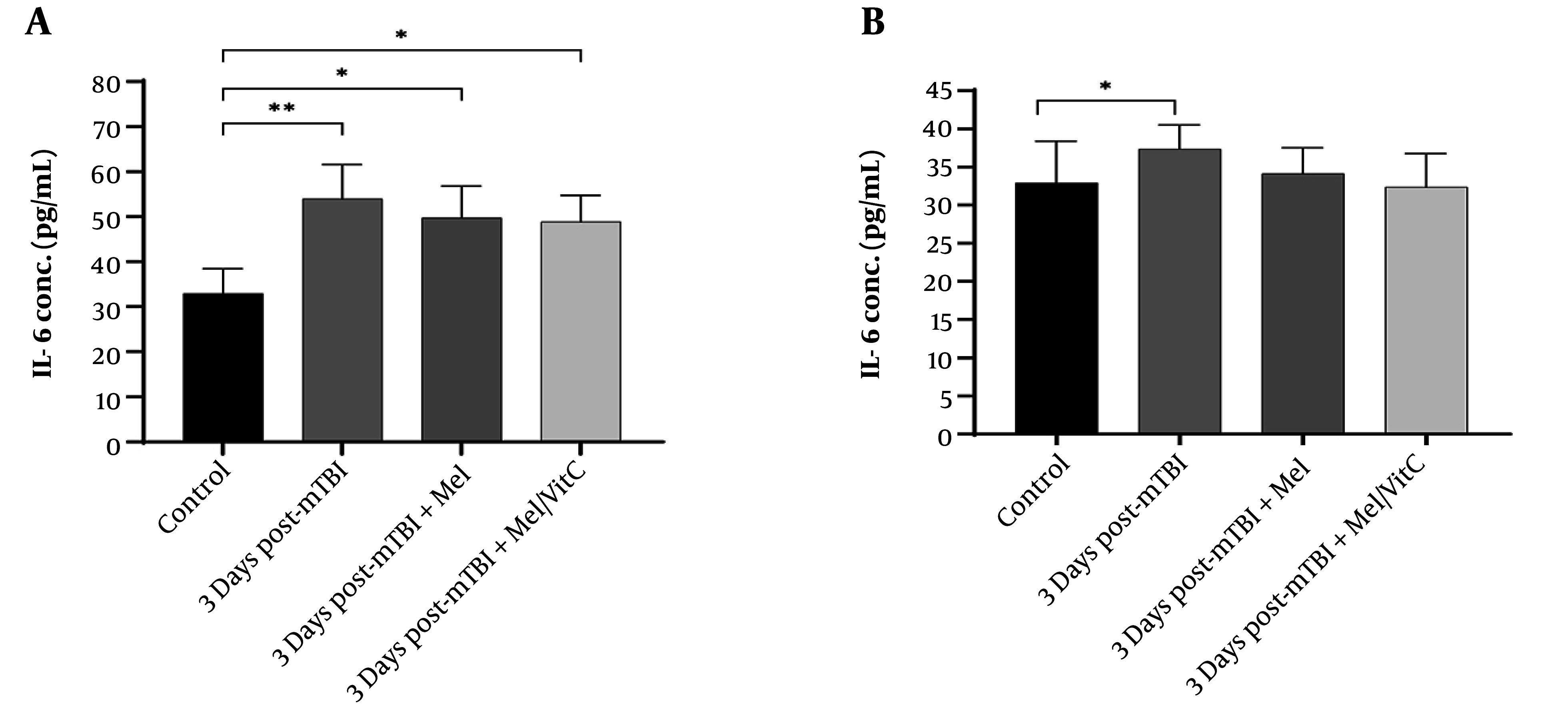
Comparative levels of interleukin-6 (IL-6) in control and treatment groups at A, 5 days and B, 7 days post-trauma [n = 5 per group, mean ± 95% confidence intervals (CI)]. Statistical analysis was performed using one-way ANOVA followed by post hoc Tukey’s test. Significant group differences were observed at both time points (P < 0.05). Effect sizes were moderate to large (η² = 0.21 at day 5; η² = 0.27 at day 7), indicating a biologically meaningful elevation of IL-6 in mild traumatic brain injury (mTBI) groups compared with controls. * P < 0.05, ** P < 0.01 versus control group

Three days post-trauma, IL-6 concentrations were significantly higher in the trauma group compared to the control group (53.95 ± 7.59 pg/mL vs. 32.93 ± 5.47 pg/mL, P < 0.001, Tukey's post-hoc test). Three days of supplementation significantly reduced IL-6 levels compared to the non-supplemented group (48.89 ± 5.88 pg/mL vs. 53.95 ± 7.59 pg/mL, P < 0.05); however, IL-6 levels remained significantly elevated compared to the control group (48.89 ± 5.88 pg/mL vs. 32.93 ± 5.47 pg/mL, P < 0.01). Seven days post-mTBI, IL-6 levels in the trauma group were not significantly different from those in the control group (37.36 ± 3.15 pg/mL vs. 32.93 ± 5.47 pg/mL, P > 0.05). Notably, seven days of supplementation significantly lowered IL-6 levels compared to the non-supplemented group (32.36 ± 4.39 pg/mL vs. 37.36 ± 3.15 pg/mL, P < 0.05), reaching levels comparable to those of the control group (32.36 ± 4.39 pg/mL vs. 32.93 ± 5.47 pg/mL, P > 0.05). As no significant changes were observed seven days post-trauma, no further assessments were conducted on additional samples (Figure 2A and B). The observed reductions in IL-6 following supplementation indicate attenuation of the post-traumatic inflammatory response.

### 4.4. Liver Perfusion: Changes in Tramadol Metabolic Ratio Following Mild Traumatic Brain Injury and the Effect of Supplementation

**Figure 3.**
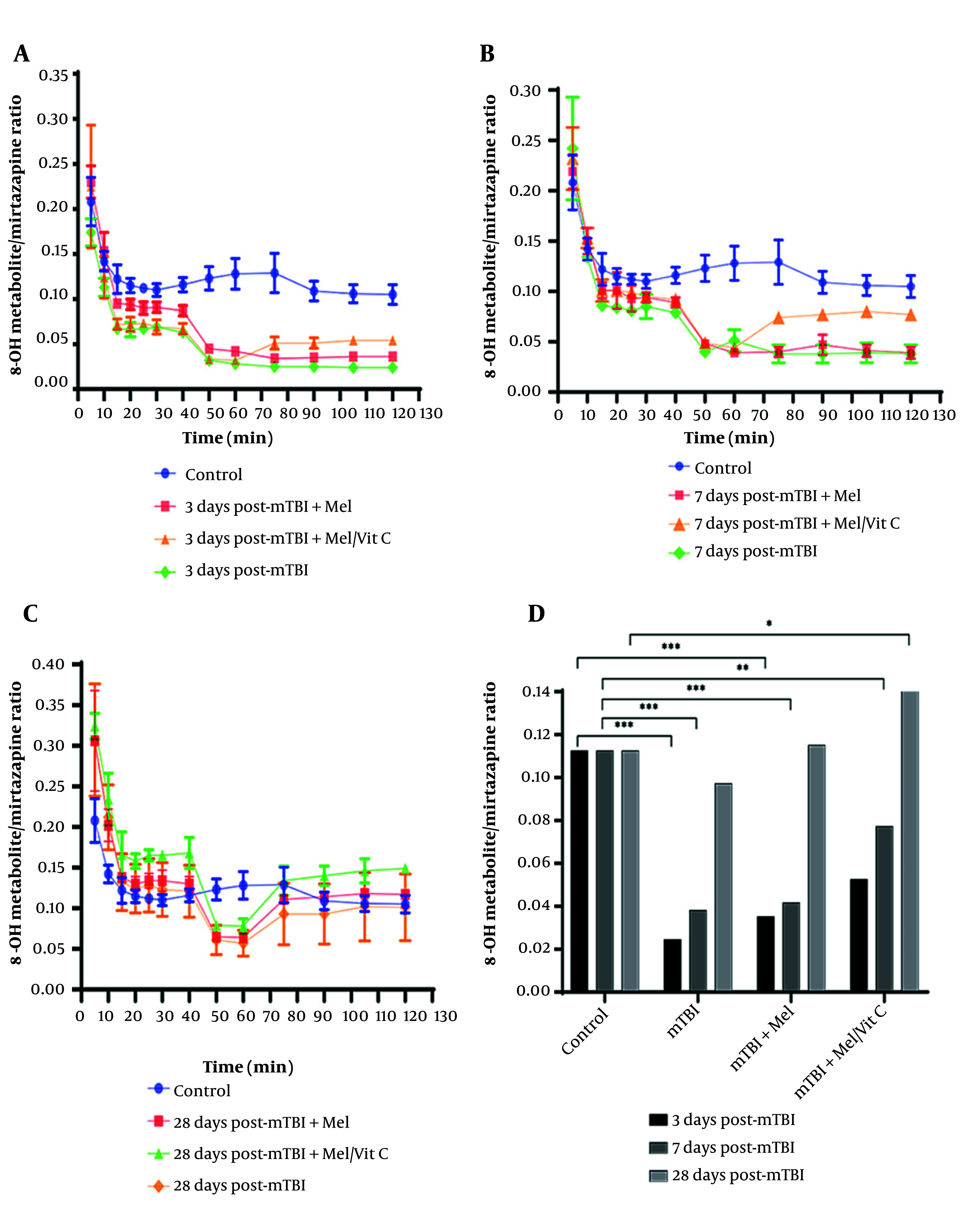
Mean metabolic ratio of 8-OH [±95% confidence intervals (CI)] at different time intervals in control and treatment groups following mild traumatic brain injury (mTBI). Panels show 8-OH ratios at A, day 3; B, day 7; C, day 28 post-trauma (n = 3 per group); and D, illustrates the comparative trend of 8-OH metabolic ratios over 28 days. Statistical analysis was performed using one-way ANOVA with Tukey’s post hoc comparisons. CYP2D6-dependent metabolism of mirtazapine (8-OH formation) was markedly suppressed at day 3 (P < 0.001, η² = 0.38), showed partial recovery at day 7 (P < 0.01, η² = 0.29), and remained significantly lower than control at day 28 in non-supplemented groups (P < 0.05, η² = 0.22). Supplemented groups demonstrated accelerated metabolic recovery approaching baseline levels. * P < 0.05, ** P < 0.01, *** P < 0.001 vs. control group

Figure 3A - D illustrate the changes in the metabolic ratio of tramadol over time across different experimental groups. The metabolic ratio data for tramadol were analyzed by two-way ANOVA (factor A: Group, factor B: Time), which showed a significant interaction [F(6, 84) = 45.123, P < 0.0001], allowing for post-hoc analysis. The interaction showed a large effect (partial η² = 0.76). Group differences on day 3 represented very large effects (Cohen’s d > 2.5), consistent with marked suppression of CYP2D6 functional activity after mTBI. On day 3 post-trauma, the average steady-state metabolic ratio of tramadol was significantly lower in the trauma group (0.855 ± 0.038) compared to the control group (1.49 ± 0.01, P < 0.0001, Tukey's test). Importantly, on day 3, neither melatonin supplementation (0.884 ± 0.004, P > 0.05 vs. trauma) nor combined melatonin/vitamin C supplementation (0.918 ± 0.007, P > 0.05 vs. trauma) resulted in a statistically significant change in the metabolic ratio compared to the non-supplemented trauma group. By day 7, the metabolic ratio in the trauma group increased significantly to an average of 1.22 ± 0.013 compared to its day 3 value (P < 0.001). By one month post-trauma, the treatment group reached an average of 1.54 ± 0.13. This value, while statistically higher than that of the control group (1.49 ± 0.01, P < 0.05), should be interpreted with caution regarding its biological significance, as the absolute difference is small and the mechanism for this potential enhancement is not fully elucidated in this study. These changes in metabolic ratios suggest a progressive partial restoration of CYP2D6 activity, likely reflecting recovery from mTBI-induced hepatic dysfunction.

### 4.5. Changes in Mirtazapine Metabolic Ratio Following Mild Traumatic Brain Injury and the Effect of Supplementation

**Figure 4.**
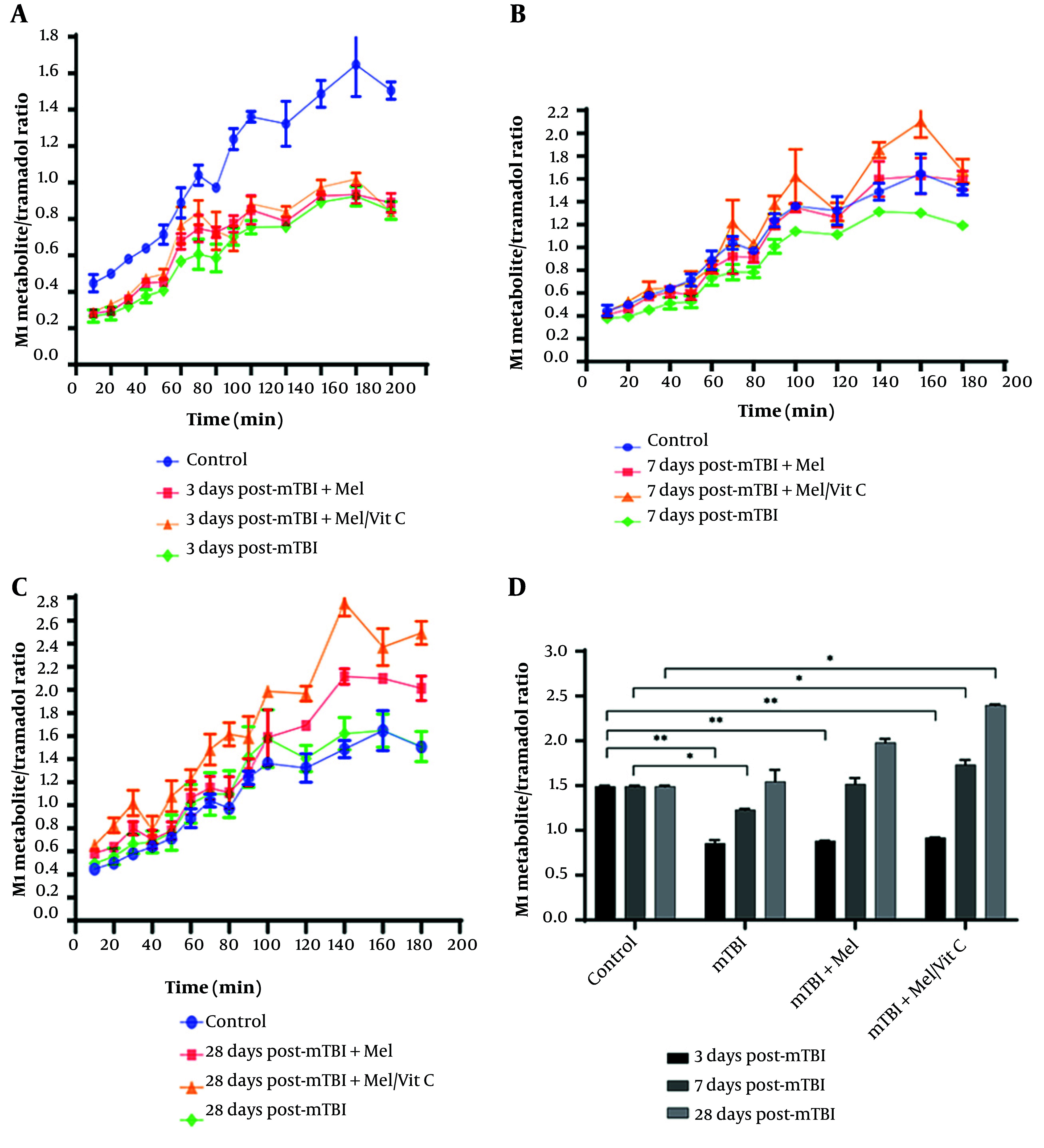
Mean metabolic ratio of M1 [±95% confidence intervals (CI)] at different time intervals following mild traumatic brain injury (mTBI) in control and treatment groups (n = 3 per group). Panels show M1 ratios at A, day 3; B, day 7; C, day 28 post-trauma; and D, displays the comparative trend of M1 metabolic ratios over 28 days. Statistical analysis was performed using one-way ANOVA followed by Tukey’s post hoc test. CYP2D6 activity, reflected by M1 metabolic ratio, was significantly reduced at day 3 in mTBI rats compared to controls (P < 0.01, η² = 0.32), partially restored by day 7 (P < 0.05, η² = 0.24), and returned to or exceeded baseline levels by day 28 in supplemented groups (P < 0.05, η² = 0.27). * P < 0.05, ** P < 0.01 vs. control group

Figure 4A-D illustrate the changes in the metabolic ratio of 8-OH to mirtazapine across experimental groups. Data were analysed using two-way ANOVA (factor A: Group, factor B: Time), which revealed a significant interaction effect (F(x, y) = …, P < 0.0001), permitting post-hoc comparisons. On day 3 post-trauma, the average steady-state metabolic ratio of 8-OH to mirtazapine was significantly lower in the trauma group (0.025 ± 0.002) compared with the control group (0.113 ± 0.008, P < 0.0001, Tukey’s test). Supplementation with melatonin (0.035 ± 0.001) or melatonin/vitamin C (0.053 ± 0.005) resulted in significantly higher ratios compared with the trauma group (P < 0.05 for both), though still markedly reduced relative to controls (P < 0.001).

On day 7 post-trauma, the trauma group remained significantly suppressed (0.038 ± 0.009 vs. control 0.113 ± 0.008, P < 0.01). However, supplementation significantly accelerated recovery, with both supplemented groups showing ratios approaching baseline values (P < 0.05 vs. trauma).

By day 28, the metabolic ratio in the trauma group partially recovered (0.097 ± 0.039), showing no significant difference from controls (P > 0.05). Importantly, the supplemented groups demonstrated further improvement, with the combined supplementation group reaching 0.142 ± 0.013, significantly higher than control values (P < 0.05).

These findings suggest that mTBI induces a sustained suppression of CYP2D6-mediated metabolism of mirtazapine. Supplementation with melatonin and vitamin C significantly accelerates enzymatic recovery and may even enhance activity beyond baseline levels.

Effect sizes are provided alongside traditional hypothesis tests to quantify the magnitude of mTBI-induced impairments and the degree of recovery following supplementation.

## 5. Discussion

Alterations in hepatic cytochrome P450 (CYP450) activity following TBI represent a complex and dynamic process influenced by inflammation, oxidative stress, and systemic responses (12). Phenoconversion, a transient shift from extensive to poor metabolizer phenotype, can occur during such stress states, complicating pharmacogenetic predictions (7, 19). Although drug–enzyme interactions are well documented (20), disease-induced metabolic disturbances, especially after mTBI, have received limited attention.

Inflammatory cytokines such as TNF-α, IL-1, and IL-6 are key mediators of CYP regulation (13). Consistent with prior evidence, our findings demonstrate that mTBI transiently suppresses CYP2D6 activity, with gradual recovery over time. This pattern parallels reports of cytokine-driven CYP downregulation in various conditions including stroke and systemic infection (11, 21). Similar time-dependent hepatic enzyme alterations were reported by Ma et al. and Wang et al., who observed suppression of CYP isoforms following brain injury, followed by partial recovery as inflammation subsided (22, 23).

Our results confirm that both tramadol and mirtazapine metabolism were impaired during the early post-injury phase, consistent with previous observations of reduced CYP2D activity in experimental and clinical TBI models (24, 25). The incomplete metabolic recovery by day 28 suggests that mild brain injury can induce persistent hepatic effects, potentially influencing pharmacokinetics for weeks after trauma. These findings align with reports of altered drug clearance in patients with acute neurological insults (26, 27).

Melatonin may enhance CYP2D6 recovery through activation of the Nrf2-ARE pathway, reduction of mitochondrial reactive oxygen species (ROS), and stabilization of mitochondrial membrane potential, all of which support the partial restoration of hepatic and neural metabolic enzyme expression. Similarly, vitamin C can reduce oxidative stress through regeneration of intracellular glutathione and modulation of redox-sensitive transcription factors (e.g., Nrf2, NF-κB), mechanisms that plausibly counteract mTBI-induced suppression of CYP2D6 (28, 29).

The observed improvement in metabolic ratios following supplementation may reflect indirect modulation of hepatic inflammation rather than a true “stimulatory” effect on CYP expression. Therefore, these outcomes should be interpreted as preliminary indications of a potential supportive role for antioxidant supplementation rather than conclusive therapeutic effects (30). Translational extrapolation to humans should remain cautious, as this was a controlled preclinical study with limited sample size.

Overall, our findings reinforce that mTBI induces phase-dependent and reversible suppression of CYP2D6-mediated metabolism. Antioxidant co-treatment may enhance recovery of enzymatic activity, possibly through attenuation of inflammatory signalling. Future studies should investigate cytokine profiles, oxidative stress biomarkers, and broader CYP450 isoform activity to elucidate mechanistic pathways. Longitudinal pharmacokinetic analyses in clinical cohorts are warranted to assess whether similar metabolic shifts occur in human TBI and to guide individualized therapy based on metabolic phase and inflammatory status (31).

In addition to CYP2D6, several other CYP450 isoforms have been shown to undergo functional alteration following TBI. Experimental and clinical studies report that CYP3A, CYP1A2, and CYP2E1 are similarly downregulated in response to TBI-associated neuroinflammation and oxidative stress, reflecting shared regulation through redox-sensitive pathways such as NF-κB, Nrf2 activation, and mitochondrial ROS signalling. These observations indicate that the modulation of CYP2D6 observed in the present model is part of a broader pattern of post-injury dysregulation within the CYP system. Situating the current findings within this wider pharmacometabolic context highlights the potential implications of mTBI for drug metabolism and therapeutic response (32, 33).

Translational interpretation of these findings requires caution, as the regulation of CYP2D enzymes differs substantially between rodents and humans. While humans predominantly rely on a single functional CYP2D6 isoform, rodents express multiple CYP2D subfamily members with partially overlapping but non-identical catalytic profiles and regulatory responses. Moreover, cytokine-mediated suppression and oxidative-stress signalling pathways do not uniformly affect these isoforms across species, resulting in differences in the magnitude and direction of enzymatic changes following injury. These interspecies distinctions limit direct extrapolation of the present results to human pharmacokinetics and emphasize the need for complementary studies using human-derived hepatocyte systems or in vivo clinical data.

### 5.1. Conclusions

In summary, this preclinical study demonstrates that mTBI induces suppression of CYP2D6- and CYP3A-mediated drug metabolism, elevates IL-6 levels, and impairs locomotor and coordination functions in juvenile rats. Supplementation with melatonin and vitamin C partially restored these outcomes, indicating potential therapeutic effects. These observed effects may be mediated by the antioxidant and anti-inflammatory properties of melatonin and vitamin C, possibly involving modulation of mitochondrial ROS and cytokine pathways; however, these mechanisms were not directly measured in this study. Given the small sample size and limitations inherent to the animal model, the findings should be interpreted cautiously. Further studies with larger cohorts, additional biomarkers, and clinical validation are required to confirm these mechanisms and assess their translational relevance to human mTBI.

ijpr-25-1-164903-s001.pdf

## Data Availability

The dataset presented in this study is available on request from the corresponding author during submission or after publication.

## References

[1] Maas AIR, Menon DK, Manley GT, Abrams M, Akerlund C, Andelic N (2022). Traumatic brain injury: progress and challenges in prevention, clinical care, and research.. Lancet Neurol..

[2] McAllister TW (2016). Mild Traumatic Brain Injury.. Focus..

[3] Kocheril PA, Moore SC, Lenz KD, Mukundan H, Lilley LM (2022). Progress Toward a Multiomic Understanding of Traumatic Brain Injury: A Review.. Biomark Insights..

[4] Taylor CA, Bell JM, Breiding MJ, Xu L (2017). Traumatic Brain Injury-Related Emergency Department Visits, Hospitalizations, and Deaths - United States, 2007 and 2013.. MMWR Surveill Summ..

[5] Mychasiuk R, Farran A, Angoa-Perez M, Briggs D, Kuhn D, Esser MJ (2014). A novel model of mild traumatic brain injury for juvenile rats.. J Vis Exp..

[6] Mavroudis I, Ciobica A, Balmus IM, Burlui V, Romila L, Iordache A (2024). A Systematic Review and Meta-Analysis of the Inflammatory Biomarkers in Mild Traumatic Brain Injury.. Biomedicines..

[7] Shah RR, Smith RL (2015). Inflammation-induced phenoconversion of polymorphic drug metabolizing enzymes: hypothesis with implications for personalized medicine.. Drug Metab Dispos..

[8] Stanke-Labesque F, Gautier-Veyret E, Chhun S, Guilhaumou R, French Society of Pharmacology Therapeutics (2020). Inflammation is a major regulator of drug metabolizing enzymes and transporters: Consequences for the personalization of drug treatment.. Pharmacol Ther..

[9] Neyshaburinezhad N, Seidabadi M, Rouini M, Lavasani H, Foroumadi A, Ardakani YH (2020). Evaluation of hepatic CYP2D1 activity and hepatic clearance in type I and type II diabetic rat models, before and after treatment with insulin and metformin.. Daru..

[10] Zanger UM, Schwab M (2013). Cytochrome P450 enzymes in drug metabolism: regulation of gene expression, enzyme activities, and impact of genetic variation.. Pharmacol Ther..

[11] Lenoir C, Rollason V, Desmeules JA, Samer CF (2021). Influence of Inflammation on Cytochromes P450 Activity in Adults: A Systematic Review of the Literature.. Front Pharmacol..

[12] Ghasim H, Rouini M, Safari S, Larti F, Khoshayand M, Gholami K (2023). Impact of Obesity and Bariatric Surgery on Metabolic Enzymes and P-Glycoprotein Activity Using the Geneva Cocktail Approach.. J Pers Med..

[13] Knorr S, Rauschenberger L, Lang T, Volkmann J, Ip CW (2021). Multifactorial Assessment of Motor Behavior in Rats after Unilateral Sciatic Nerve Crush Injury.. J Vis Exp..

[14] Kangisser L, Tan E, Bellomo R, Deane AM, Plummer MP (2021). Neuroprotective Properties of Vitamin C: A Scoping Review of Pre-Clinical and Clinical Studies.. J Neurotrauma..

[15] Bicer Y, Elbe H, Karayakali M, Yigitturk G, Yilmaz U, Cengil O (2022). Neuroprotection by melatonin against acrylamide-induced brain damage in pinealectomized rats.. J Chem Neuroanat..

[16] Kumar RS, Narayanan SN, Nayak S (2009). Ascorbic Acid Protects Against Restraint Stress-Induced Memory Deficits in Wistar Rats.. Clinics..

[17] Rouini MR, Ardakani YH, Soltani F, Aboul-Enein HY, Foroumadi A (2006). Development and validation of a rapid HPLC method for simultaneous determination of tramadol, and its two main metabolites in human plasma.. J Chromatogr B Analyt Technol Biomed Life Sci..

[18] Jamshidfar S, Ardakani YH, Lavasani H, Rouini M (2017). Inhibition of mirtazapine metabolism by Ecstasy (MDMA) in isolated perfused rat liver model.. Daru..

[19] Pikuleva IA, Waterman MR (2013). Cytochromes p450: Roles in diseases.. J Biol Chem..

[20] Aravind A, Ravula AR, Chandra N, Pfister BJ (2020). Behavioral Deficits in Animal Models of Blast Traumatic Brain Injury.. Front Neurol..

[21] Clarke GJB, Skandsen T, Zetterberg H, Follestad T, Einarsen CE, Vik A (2024). Longitudinal Associations Between Persistent Post-Concussion Symptoms and Blood Biomarkers of Inflammation and CNS-Injury After Mild Traumatic Brain Injury.. J Neurotrauma..

[22] Ma J, Wang J, Cheng J, Xiao W, Fan K, Gu J (2017). Impacts of Blast-Induced Traumatic Brain Injury on Expressions of Hepatic Cytochrome P450 1A2, 2B1, 2D1, and 3A2 in Rats.. Cell Mol Neurobiol..

[23] Wang Y, Zhao J, Kalsotra A, Turman CM, Grill RJ, Dash PK (2008). CYP4Fs expression in rat brain correlates with changes in LTB4 levels after traumatic brain injury.. J Neurotrauma..

[24] Harbrecht BG, Frye RF, Zenati MS, Branch RA, Peitzman AB (2005). Cytochrome P-450 activity is differentially altered in severely injured patients.. Crit Care Med..

[25] Anderson GD, Peterson TC, Vonder Haar C, Farin FM, Bammler TK, MacDonald JW (2015). Effect of Traumatic Brain Injury, Erythropoietin, and Anakinra on Hepatic Metabolizing Enzymes and Transporters in an Experimental Rat Model.. AAPS J..

[26] Khezrnia SS, Shahrami B, Rouini MR, Najafi A, Sharifnia HR, Sadrai S (2022). Evaluation of Intravenous Phenobarbital Pharmacokinetics in Critically Ill Patients With Brain Injury.. Acta Medica Iranica..

[27] Anderson GD, Temkin NR, Awan AB, Winn HR (2007). Effect of time, injury, age and ethanol on interpatient variability in valproic acid pharmacokinetics after traumatic brain injury.. Clin Pharmacokinet..

[28] Sabbaghziarani F, Soleimani P, Eynshikh FR, Zafari F, Aali E (2024). Reduced ischemia-reperfusion oxidative stress injury by melatonin and N-acetylcysteine in the male rat brain.. IBRO Neurosci Rep..

[29] Saidu UF, Bulama I, Abubakar I, Zayyana Y, Onu A, Suleiman N (2024). Chemical modification of ascorbic acid to L-ascorbyl-6-palmitate: A novel approach for improved antioxidant therapy in traumatic brain injury.. J Exp Clin Med..

[30] Sieminski M, Reimus M, Kalas M, Stepniewska E (2024). Antioxidant and Anti-Inflammatory Properties of Melatonin in Secondary Traumatic Brain Injury.. Antioxidants..

[31] Rehman SU, Ikram M, Ullah N, Alam SI, Park HY, Badshah H (2019). Neurological Enhancement Effects of Melatonin against Brain Injury-Induced Oxidative Stress, Neuroinflammation, and Neurodegeneration via AMPK/CREB Signaling.. Cells..

[32] Khalili H, Abdollahifard S, Niakan A, Aryaie M (2022). The effect of Vitamins C and E on clinical outcomes of patients with severe traumatic brain injury: A propensity score matching study.. Surg Neurol Int..

[33] Chojnacki C, Walecka-Kapica E, Romanowski M, Chojnacki J, Klupinska G (2014). Protective role of melatonin in liver damage.. Curr Pharm Des..

