# Supplementary material for: Impact of Mild Traumatic Brain Injury (mTBI) on CYP2D6 Activity and the Restorative Effects of Melatonin and Vitamin C Supplementation
Source: Iran J Pharm Res. 2026 Feb 8;25(1):e164903. doi: 10.5812/ijpr-164903 (PMC13180301; doi:10.5812/ijpr-164903)
Supplement: ijpr-25-1-164903-s001.pdf [file ijpr-25-1-164903-s001.pdf]

Supplementary file

Run	Block	Factor 1: A: Force	Factor 2: B: Weight	Factor 3: Helmet	Response R1: NSS Score (mean±SD)	Response R2: Time to right (min)
1	Block 1	1.83	194.19	Yes	4±0.7	44
2	Block 1	0.45	150.00	No		
3	Block 1	0.75	250.00	No		
4	Block 1	0.45	250.00	Yes		
5	Block 1	3.20	216.87	Yes		
6	Block 1	1.48	150.00	Yes	3±0.6	28
7	Block 1	3.20	150.00	No		
8	Block 1	0.45	200.00	No		
9	Block 1	0.45	250.00	Yes		
10	Block 1	1.83	187.47	No		
11	Block 1	3.20	150.00	Yes	6±1.11	101
12	Block 1	0.45	150.00	No		
13	Block 1	3.20	250.00	No		
14	Block 1	3.20	250.00	Yes		
15	Block 1	1.83	250.00	No		
16	Block 1	3.20	200.00	No		
17	Block 1	3.20	216.78	No		
18	Block 1	0.45	150.00	Yes	2±1.21	24
19	Block 1	0.45	183.39	Yes	1±0.9	15

Appendix.1 Experimental Design table for Induction of Mild Traumatic Brain Injury

**Task**	**Points**
Presence of mono- or hemiparesis	1
Inability to walk on a 3-cm-wide beam	1
Inability to walk on a 2-cm-wide beam	1
Inability to walk on a 1-cm-wide beam	1
Inability to balance on a 1-cm-wide beam	1
Inability to balance on a round stick (0.5 cm diameter)	1
Failure to exit a 30-cm-diameter circle (for 2 min)	1
Inability to walk straight line	1
Loss of startle behavior	1
Loss of seeking behavior	1
Maximum total	10

Appendix 2 The 10-point Neurological Severity Score table
